# Soil, Hand, and Body Adherence Measures across Four Beach Areas: Potential Influence on Exposure to Oil Spill Chemicals

**DOI:** 10.3390/ijerph17124196

**Published:** 2020-06-12

**Authors:** Alesia Ferguson, Ashok Kumar Dwivedi, Esther Ehindero, Foluke Adelabu, Kyra Rattler, Hanna Rose Perone, Larissa Montas, Kristina Mena, Helena Solo-Gabriele

**Affiliations:** 1 Department of Built Environment, North Carolina Agricultural and Technical State University, Greensboro, NC 27411, USA; akdwivedi@ncat.edu (A.K.D.); eehindero@aggies.ncat.edu (E.E.); fradelabu@aggies.ncat.edu (F.A.); 2 School of Social Work, University of Arkansas Little Rock, Little Rock, AR 72204, USA; kyra.brown11@yahoo.com; 3 Department of Civil, Architectural and Environmental Engineering, University of Miami, Coral Gables, FL 33146, USA; hrp34@med.miami.edu (H.R.P.); l.montas@umiami.edu (L.M.); hmsolo@miami.edu (H.S.-G.); 4 School of Public Health, University of Texas-Houston, El Paso, TX 79905, USA; Kristina.D.Mena@uth.tmc.edu

**Keywords:** hand adherence, body adherence, children’s exposures, beach exposures, risk of exposure to oil spill contaminants

## Abstract

Skin adherence (SA) of soil affects exposure from soil contaminants through dermal routes via loading on the skin and through ingestion routes through hand to mouth activities. The objectives of this study were to evaluate the relationships between adherence versus child-specific and environmental factors. Two sets of soil-to-skin adherence were evaluated. The first was based on loading on hands following hand presses (Hand SA). The second was based on body rinses following one hour of play activities on the beach (Body SA). Results for 98–119 children conducted at four beach sites show that mean Hand SA was 35.7 mg/cm^2^ (std. dev. 41.8 mg/cm^2^), while Body SA based on full coverage was 6.8 mg/cm^2^ (std. dev. 4.8 mg/cm^2^). Statistically significant differences in Body SA were observed between male (avg. 8.1 mg/cm^2^) and female (avg. 5.8 mg/cm^2^) children (*p* < 0.05). No significant difference by sex was found for Hand SA. Other statistically different observations were that Hand SA (*p* < 0.05), but not Body SA, differed across the four beaches (*p* < 0.05). For Hand SA, this difference was associated soil size variability across the beaches. Hand and Body SA values measured during this study are recommended for use in risk assessments that evaluate beach exposures to oil spill chemicals for young children.

## 1. Introduction and Background

Children, compared to adults, are more vulnerable to adverse environmental exposures for a number of reasons. The unique ways in which they interact with their environment may mean they receive a higher dose of toxicant for a given level of environmental contamination compared to adults. They consume more food and water and have higher inhalation rates per pound of body weight than adults. Young children also play close to the ground and come into contact with contaminated soil outdoors and with contaminated dust on surfaces and carpets indoors [1]. Studies show that children display unique activity patterns during their play activities, such as frequent hand and mouth contact behaviors with objects/surfaces and even ingestion of soils [2,3,4,5,6,7,8,9,10]. In addition, children can suffer greater adverse health outcomes from exposures depending upon the timing of exposure with developmental stage [11,12]. Accurately estimating their exposures is important for ultimately determining doses and health outcomes. Exposure relies on knowledge of activity patterns (e.g., contact rates, breathing rates, time spent in microenvironments), along with environmental concentrations.

There are various exposure concerns at beaches, where environmental pollution (i.e., presence of bacteria and chemical pollutants) may be present in sand and water. Metals from various natural and anthropogenic sources can accumulate in soil and on surfaces [13,14]. In addition, children can be exposed to other contaminants, such as polycyclic aromatic hydrocarbons (PAHs) [15,16,17] and phthalates [18,19,20], in soils and/or dusts via dermal and ingestion exposure both on beaches and in and around homes. At beaches specifically, sand play or swimming can potentially cause illnesses like stomach ailments, fever, and other acute or chronic health outcomes [21,22], where in addition to ingestion and sorption through skin exposures can also occur through open wounds or eyes. Soil–skin adherence is defined as the adherence of any particle-matrix (e.g., sand, dirt, soil) to the skin. Soil adherence values help in the estimation of exposure to environmental pollutants found in sands. 

That latest edition of the Exposure Factors Handbook was published in 2011 for exposure related data on children and adults [23]. This book provides the latest factors used in the assessment of exposures in humans, to include for example, consumption of drinking water and foods, soil ingestion, inhalation rates, dermal factors (e.g., skin area and soil adherence factors), consumer product use, and building characteristics. This book includes recommended values for the general populations and also for specific segments of populations. The EPA has released updates on individual chapters since October 2017. The National Center for Environmental Assessment (NCEA) also published the Child Specific Exposure Handbook in 2008, which includes exposure factors and related data to facilitate the need to consolidate all child exposure data into one document [1]. This child-specific handbook also provides equations for children’s assessment of inhalation, ingestion and dermal exposures. The data contained however has limited information for field studies conducted at beaches.

Even though it is possible to control and assess the main factors that may affect soil adherence to skin in laboratory experiments [24,25,26,27], it is difficult to simulate real-world conditions involving serial contact events or typical activities of children in the field. To date, there have been some field studies that investigated soil-to-skin adherence factors [28,29,30,31], but measures are limited at beach settings. Previous studies indicated that soil loadings in other settings (e.g., home) varied by body part and type of activity and, in general, were higher for hands and feet than for forearms and lower legs [28,29,30]. Studies also reported that soil loadings for hands were dependent on the types of activity performed. Yamamoto et al. [31] reported that the mass and size distribution of soils that adhered to hands of children largely varied with the play patterns, even though the children played in the same area. In general, the number of children studied was small (*n* < 10) in the same or similar activity category across these previously published studies. Other studies conducted in laboratory settings used adult volunteers to determine soil adherence under different affecting factors. However, the number of subjects was also small (*n* of subjects = 1–10) in these laboratory settings [27,31,32]. 

Larger field studies of natural play can provide realistic estimates of soil adherence to different body parts by children contacting soils. The principal objective of this study was to evaluate the soil-to-skin adherence for children under seven years of age based on the field measures across four major beaches (two in Miami, FL, USA and two in Galveston, TX, USA) involving 122 children after they had engaged in beach play outdoors. We estimate the soil-to-skin adherence based on two measures: (1) Loading on hands following hand presses and, (2) body rinses following one hour of play activities on the beach, where we looked at the effect of age, gender, sunscreen and other variables on these soil-to-skin adherence values. The soil adherence (SA) values for hands (Hand SA) has been previously published [33]. This paper will present the collection of body soil adherence (Body SA) values for the first time, along with a comparison between Hand SA and Body SA values across the 122 children. These adherence measures were part of a larger study to evaluate children’s behavior that might influence exposures to oil spill contaminants (OSC) that might be found at beaches. The larger study entailed the collection of child and parental demographics, child clothing coverage, videography data, beach profile characteristics, and soil and environmental conditions [34,35]. Soil adherence is a measure of mass of soil adhered per area of skin. Again, soil adherence factors are useful not only for dermal estimate but are used to estimate soil ingestion rates for children [6,8].

## 2. Methods

**Figure 1:**
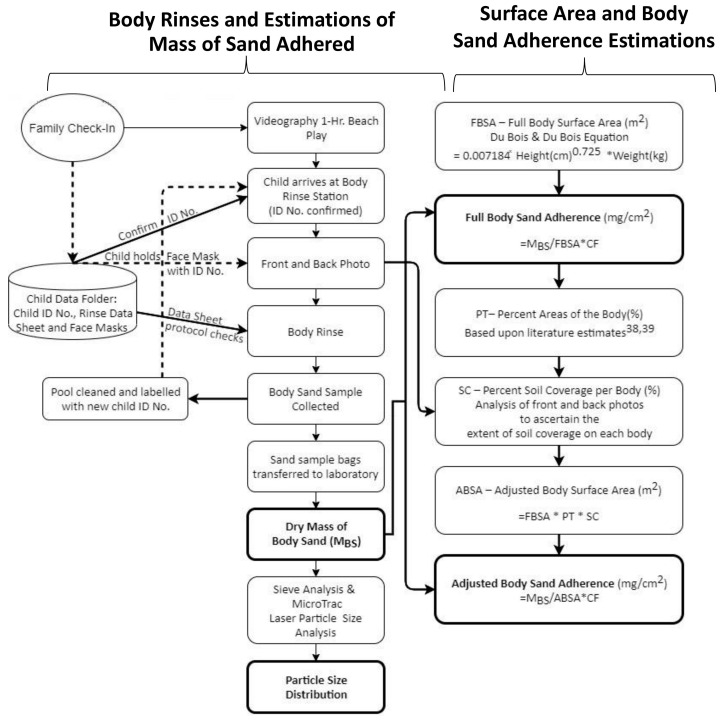
Flow chart providing a graphical representation of the methods utilized in this study. More details for the process for each measured value are provided in the Methods section. Bold boxes show the measured values; see Results section for value summary and statistical analysis.

SA values (mg/cm^2^) were based on staged hand press trials and body rinses following beach play, where Figure 1 is a flowchart of the procedures and methods. This SA normalized parameter is useful for comparison across studies and used in dermal and ingestion exposure estimates. Discussed below are the subjects and location (emphasizing relevance to possible influence on the SA value), procedure for performing the hand press trials and body rinses, collection of other potentially influential variables (e.g., last play activity as determined by video, clothing), determination of hand and body surface areas (i.e., area values used to normalize the mass of sand adhered), determination of Body Sand Adherence values and the statistical routines performed. IRB protocols were approved and established for this project through the University of Miami (IRB 20140140-MOD00023226) and University of Texas (IRB #HSC-SPH-18-0396). 

### 2.1. Subjects and Beach Locations

There were 125 children originally enrolled in the field study. Inclusion criteria for children was that they were six years old or less, that they were able to walk and participate in active play activities at the beach, and that their guardian was capable of providing consent to participate in the study. Given this criteria, data for three children were removed due to parental lack of consent, non-age compliance, and non-adherence to field task. Sixty-two children were enrolled in Miami and 60 children were enrolled in Texas for hand press trials and body rinses all under the age of seven years. Given the inclusion criteria of the child’s ability to walk, only one subject under 1 year of age was recruited. Hand presses and body rinses were conducted at four different beach locations. The two beaches in Miami, Florida were Crandon (25°42′1″ N, 80°09′14″ W) and Haulover (25°54′03″ N, 80°07′19″ W) and the two beaches in Galveston, Texas were Stewart (29°18′24″ N, 94°45′56″ W) and Seawall (29°16′12″ N, 94°49′7.4″ W). Seawall Beach is also referred to as Urban Park Beach. The criteria for choosing the beaches was based upon geographic distribution and geomorphology. The beaches were in two geographically different regions (within two different U.S. States). Within a geographic region, beaches were chosen based upon different geomorphology that could influence play behavior. Within each region, the geomorphology of one beach (either Stewart Beach and Crandon Beach) was conducive to child play with a very wide beach (150 to 160 meters) and shallow intertidal zone (0.012 m/m) with low wave heights (<0.7 m on average). The second beach in Galveston (Seawall Beach) was also shallow but had a number of rock groins to minimize beach erosion which may have influenced child play behavior. The second beach in Miami (Haulover Beach) had a slightly higher average wave height (1 m) and steeper slope (0.024 m/m) which we anticipated could also have impacted child play behaviors. This beach also had a rock groin that was close to where the children played. At the time of the study, Crandon was covered by seaweed close to shoreline, and has tidal changes influencing the presence of sand bars. Haulover Beach had steeper sloping sand due to recent storm erosion.

### 2.2. Hand Press Procedures

Sand was collected where children played the most, from the upper surface of the supratidal zone (about 1 cm depth) using a shovel and placed in a bucket for use throughout the day. Subsequently, hand press trials were performed by instructing the children to place their palm on a sand laden tray for five seconds. The weight was then recorded (by a scale placed under tray) and converted to pressure of contact (psi). The hands of the children were then rinsed in a plastic zip-top bag filled with sediment free ocean water (one liter) for removing the sand that adhered to hands. The children were randomly selected for testing their hands under one of three conditions (wet, dry and sunscreen). Further details on the hand press trials and the detailed results for Hand SA can be found in Ferguson et al. [33].

### 2.3. Body Rinse Procedures

A body rinse station was located under a tent, approximately 15 m inland from the supratidal zone where the majority of children played. Each body rinse site was set-up with a flexible plastic wading pool, 209 cm (59 inches) in diameter and two plastic watering cans. The watering can’s spout had a shower head 15 cm (4.2 inches) in diameter, and were pre-filled to full capacity, 7.6 L (2 gallons), with sediment free ocean water collected approximately 6 m beyond the swash zone. In accordance with the data quality control procedures for this study, a child data folder clearly identified the child ID and contained the field data sheets and face masks for each child. Children were rinsed immediately after they were videotaped for one hour of play on the beach, with any clothing and shoes (if worn) used during their play activity. To record the areas of body covered with sand adhered and the clothing worn, a photo was taken front and back. For privacy purposes, the child covered their face by holding the paper mask, which included the child ID. The body rinse data sheet was used to record notes detailing the use or reapplication of sunscreen on the body and the type of clothing worn. 

Upon arrival of the child to the body rinse site, the pool was inspected again to ensure it was free of sand. All researchers were careful not to accidentally move sand into the pool during the body rinse. The child was asked to step into the center of the pool or was carried in by the parent (if young) and the parent was asked to rinse the child. Each watering can was held about 20 cm above the child’s body and water was allowed to fall freely from the showerhead at a steady flow, starting over the head including the hair and subsequently over the whole body. If the whole body was rinsed and there was water remaining in the can, the rinse continued over the head again and moving downwards. When the first watering can was emptied, the process was repeated using the second watering can. The child was then asked to step out of the pool or was carried out by the parent. Notes were taken to account for any incident for noncompliance with the protocol and the rinse data sheet was placed in the child information folder before proceeding to the check-out station.

The pool water was left to settle for 10 min. The excess water was then decanted carefully to prevent sediment resuspension. The pool was then lifted off the ground, tilted slightly sideways, flexible sides pressed together to create channel and shaken energetically in a circular motion in such a way that the sediment grouped together in one area. As the pool was tilted further, the remaining water and sediment flowed into a pre-labelled and pre-weighted plastic zip-top bag. While the pool and zip-top bag remained in the same position, a squirt bottle with sediment free ocean water was used to rinse off the sides of the pool to collect in the bag any sediment that had adhered to the sides of the pool. The pool was cleaned with a wipe, dried with a clean towel and labelled for the next child in the study. The watering cans were rinsed and refilled with sediment free ocean water. The zip-top bags were held stationary for 10 min to allow the sediments to settle into one corner of the bag. Excess water was then decanted carefully until the water flowed without disturbing the sediment. The bags with the sediment samples were secured to avoid accidental spillage and placed in a cooler, on ice in the dark to slow down the growth of microorganisms. At the end of each day, both the sediment samples from hand rinse and body rinses were then transferred from the cooler to a refrigerator. Posteriorly, the samples were transported to the University of Miami where total mass and particle size distribution analyses were conducted.

### 2.4. Determination of Mass and Size of Sand Particles Adhered to Hands and Body

Upon processing at the laboratory, individual sand samples from the hand press trials and body rinses were processed by repeating the field decanting of water by orienting the zip-top bags with the corner containing the sediments downwards. After about 30 min, the sediments were again decanted to remove as much of the overlying water as possible without losing the sediment. Once decanted, the corner of the zip-top bag was then cut about a centimeter above the sediment line. The sediment was then transferred to a pre-weighed aluminum tin using a scoopula and then rinsed with de-ionized water to transfer residual sediment. The aluminum tins with the sediment were then placed in an oven set to 100 °C for 24 h. After drying, the tins with the dry sediments were then reweighed, and the mass of sediment (M_s_) was determined from the difference of the weight of the tin with dry sediment and the weight of the tin.

For each child, particle size distributions from hand and body rinses were determined using a Microtrac laser diffraction particle size analyzer (S3500), for samples that weighed more than 0.1 g. To avoid clumping, samples were washed three times in isopropyl alcohol and de-ionized water through a sequence of washing and decanting steps minimizing any soil loss. Given that the maximum size measurable by the Microtrac analyzer was 1200 µm, particles larger than 1180 µm were removed by sieving. The masses of sediment captured on the sieve and the amount that went through the sieve were recorded. Mean particle diameters were adjusted to account for the larger particles that did not pass through the Microtrac analyzer. Ambient sediments were measured for particle size distribution in a similar manner. For comparison of each child SA measure, the average ambient grain size for that beach was matched. Further details can be found in Ferguson et al., 2020 [33].

### 2.5. Surface Area Measurements

***Hand*:** Hand surface area in cm^2^ were used to calculate mass of sand adhered per unit area of hands during hand press trials. In order to trace the hand area, children were asked to place their hands on a graph paper having 0.1 cm × 0.1 cm grid. Following the tracing, researchers used the software program ImageJ (https://imagej.nih.gov/ij/) to calculate area of left hand and right hand for each child by scaling graph paper boxes and manually digitizing the area for each hand. Further details on the estimate of hand surface area measures can be found in Perone et al. [36] including a comparison to other techniques that used children height and weight for the calculation of hand surface area.

***Body*:** There were two approaches taken to estimate the area needed for body SA values: (1) Full-Body surface area (FBSA) which is defined as the mass adhered to the body normalized over the total body surface area, and (2) Adjusted-Body surface area (ABSA) which is defined as the mass adhered to area of skin normalized by area of body where soil appeared as adhered in field photos. The Full-Body surface area (FBSA) was calculated using Du Bois and Du Bois Formula [37]:

$$\mathrm{BSA}\;\left(m^{2}\right)=0.007184\times \mathrm{Height}\;{\left(\mathrm{cm}\right)}^{0.725}\times \;\mathrm{Weight}\;{\left(\mathrm{kg}\right)}^{0.425}\;$$

For adjusted surface area calculations, following sand play for approximately one hour, researchers used the front and back photos of the children prior to body rinses. Based on the photos, researchers recorded whether sand appeared to adhere to various parts of the body. Based on literature estimates of fractional areas of the body with respect to the total surface area [38,39], percentages of FBSA (PT) were assigned as follows: chest and upper back were estimated as 18%, front and back of legs were estimated as 18% each, (front and back of feet were estimated as 4% of that 18% for each leg), front and back of arms and hands were estimated as 18% for both and abdomen and lower back was estimated to also be 18%. The head and neck were assigned as 9% but ignored here due to coverage of children’s faces. Researchers also assigned numbers 1, 2 and 3 to ascertain the extent of soil coverage on these body parts, where 3 represents no soil coverage (SC) (0% coverage), 2-partially covered (50% coverage), and 1- fully covered (100% coverage). Therefore, the area of the body covered with sand was estimated by the sum of all FBSA × PT × SC.

### 2.6. Body Soil Adherence Calculations

Full-body sand adherence was determined as the mass of soil adhered (M_s_) normalized over the Full-Body surface area (FBSA). Adjusted-Body sand adherence was determined as the mass of soil adhered normalized over the Adjusted-Body surface area (ABSA). A conversion factor (CF) was used for both parameters to convert FBSA and ABSA units to cm^2^.

### 2.7. Collection of Ambient Sediment Samples in the Field and Environmental Parameters

Sediment samples of about 2 kg per sample were collected at the beginning of each study day to characterize them for particle size and mineralogy (calcium carbonate fraction). Ambient sand was collected by scraping the top centimeter of sediments from the supratidal area near the beach shore in the vicinity of where children would play. Ambient sand samples were placed in a zip-top bag and were placed on ice in the dark in the field and then in a refrigerator at the end of each sample collection day until processing for analysis immediately after the Miami or Galveston study days. Average grain size diameter measured across the beaches over 3–4 days were (1) Crandon = 348.9 microns, (2) Haulover = 796.7 microns, (3) Stewart = 162.2 microns, and (4) Seawall was 165.7 microns. In addition, the percent calcium carbonate for the Miami beaches ranged from 40.3 to 90.4% and for the Texas beaches from 1.3 to 4.5% for samples collected during the days of the study. Environmental data was collected for modeling their association with various sand adherence measures. A multi-probe meter (YSI Model 650, Yellow Springs, OH, USA) was used to measure water temperature and salinity. Additionally, a laser thermometer (Raytek Minitemp, Fluke Corporation, Everett, WA, USA) was used to measure the temperature of the sand. Other weather conditions like air temperature, wind speed, visibility, and humidity were recorded by NavClock (Microsoft Apple Application v3.3.5, Redmond, WA, USA). 

### 2.8. Statistical Analysis

A number of statistical analyses were used to compare measures of Hand SA and Body SA, and their relationship to other variables like adhered grain size, ambient grain size, ambient humidity, water temperature, sand temperature and air temperature, clothing worn, sunscreen application, and last play activity before rinsing. The comparison of Hand SA to Body SA is of interest as decisions are made on the appropriate standardized soil-to-skin adherence value to be used in models estimating dermal or ingestion exposure and dose. The distribution of values are first presented by comparing the means across groups (i.e., females/males, age groups, beach locations) for soil adherence estimates and then evaluating whether the mean for one group differed significantly from the mean of another group using a two samples t-test. In cases where the distributions of the sand adherence estimates have unequal variances the Satterthwaite method was used, otherwise the pooled method was used for equal variance. Linear regression analysis was also used to explore the relationship of independent variables to Hand SA, Full-Body SA, or Adjusted-Body SA values.

The analysis of variance (ANOVA) method was used for testing the significant differences where more than two groups were involved in hypothesis testing. Although ANOVA indicates significant differences among more than two groups, it does not provide specifics about the differences in a pair of groups. Tukey’s Test was used for post-hoc analysis, which compared the means of all groups to every other groups and identified pairs that were significantly different. Some data are presented through box and whisker plots with outliers shown by circles, high and low whisker error bars show outer 1.5 interquartile ranges, box edges representing 25th and 75th percentiles, the circle in the box representing mean, and the line within the box representing median. Some data are also presented in fit plots, where the shaded area represents the 95 confidence limits for the data and the line represents the prediction limits that encompass more scatter and uncertainty in future data. A total of 98 measures were used for Hand SA due to the removal of 24 measures found to have limitations. A total of 119 measures were used for Full-Body SA after removing 3 cases for which a mass soil was not available. For Adjusted-Body SA, 108 measures were available based on the additional unavailability of pictures for adjustment.

## 3. Results

Results are presented below for comparisons between Hand SA and Full- and Adjusted-Body SA measures. This is followed by evaluating how SA measures vary by sex, age groups, and beach locations. Of interest was a determination of what other variables influence SA measures, such as environmental conditions of temperature and humidity and the average size of sand particles that have adhered to the children’s skin or found at the beach locations. Other variables were explored for their effect on SA and include the last location and last activity of the child as observed in the video and the clothing worn by the child. This study offered a wealth of data from other domains (e.g., videotaped data and field notes), and therefore correlations were also explored between SA measures and clothing types, last location and activity from the videotapes. The predominance of findings on Hand SA were submitted for publication [33] only briefly presented here for comparison to Body SA measures.

### 3.1. Previous Findings on Hand SA

Our previously published paper presented findings for Hand SA data for 98 children following exclusion of data for 24 children based on errors or limitations [33]. Some of the main findings are presented here. For the 45 male and 53 female children, there was no significant difference in Hand SA and pressure of contact by sex, although significant differences in the variance for Hand SA was observed (F = 2.78, *p* =0.0005). Similarly, in a pairwise t-test, Hand SA across three age groups (0–24 months, 25–48 months and >48 months) showed higher Hand SA in younger children than older. However, no significant differences were observed in means although significant differences were observed in variance. Pooled t-test for equal variance and Satterthwaite test for unequal variance showed that Hand SA for children grouped by beaches was statistically significantly different for the Miami beaches (Crandon, Haulover) versus the Texas (Stewart, Seawall). In addition, Hand SA differed significantly based on dry versus wet and sunscreen versus wet condition of testing. Linear regression also showed that the average adhered grain size and average ambient grain size had a statistically significant influence on Hand SA (both *p* < 0.0001), where the ambient soil size and condition of testing was able to explain 45% of variance in Hand SA.

#### Summary of Variables for Hand and Body Adherence

Results (Table 1) show that the mean for Full-Body SA was over 5 times less than Hand SA, while Adjusted-Body SA was over 10 times that of Full-Body SA. For the 122 studied children under age seven years, mean body weight was 16.76 kg (37.24 lbs) while mean height was 100.83 cm (39.70 inches).

### 3.2. Hand and Body (Full and Adjusted) SA by Sex

**Figure 2:**
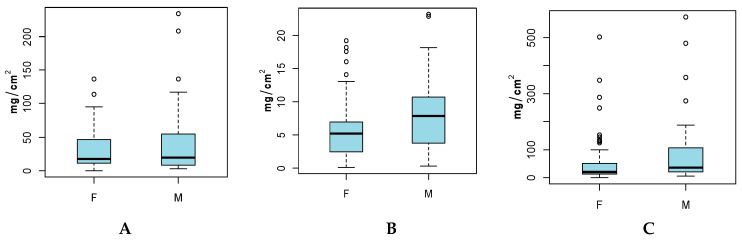
(**A**) Hand SA (mg/cm^2^), (**B**) Full-Body SA (mg/cm^2^) and (**C**) Adjusted-Body SA (mg/cm^2^) by Sex. Grey points indicate outliers.

Results comparing males to females (Figure 2 and Table 2) show that male children had higher SAs than female children for all three SA categories. For Hand SA and Adjusted-Body SAs that difference in means was not significant, whereas for Full-Body SAs there was a significant difference in means for female and male children (*p* = 0.0113). Significant differences were observed for the variances of Hand SA and Adjusted-Body SA between females and males.

### 3.3. Hand and Body (Full and Adjusted) SA by Age Group

**Figure 3:**
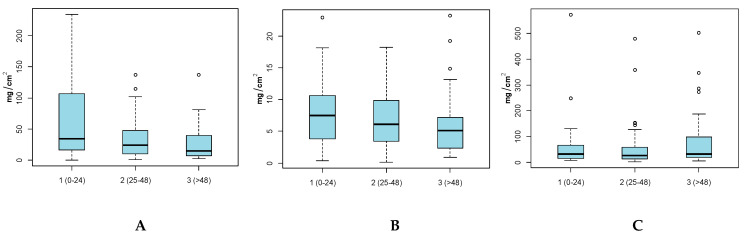
(**A**) Hand Soil SA (mg/cm^2^), (**B**) Full-Body SA (mg/cm^2^) and (**C**) Adjusted-Body SA (mg/cm^2^) by Age Groups. Grey points indicate outliers.

Results by age group (Figure 3) show that the youngest age groups of children, group 1 (0–24 months), had higher SAs with respect to older age groups. For example, the mean Hand SA for the youngest child group was 77.4 mg/cm^2^, which was higher than group 2 (25–48 months) and 3 (>48 months) (34.4 and 27.8 mg/cm^2^ respectively). Based on the two sample student’s t-test, this difference was not found to be statistically significant (alpha = 0.05). Significant differences in means were observed among age group 1 and 3 (*p* = 0.0462) for Full-Body SA (Table 3). Additionally, no significant difference in means for Adjusted-Body SA was observed for the three age groups. 

### 3.4. Hand and Body (Full and Adjusted) SA by Beach Locations 

**Figure 4:**
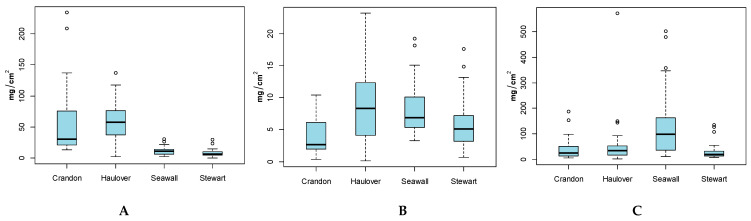
(**A**) Hand SA (mg/cm^2^), (**B**) Full-Body SA (mg/cm^2^) and (**C**) Adjusted-Body SA (mg/cm^2^) by Beach Location. Grey points indicate outliers.

Results across beaches (Figure 4 and Table S1) show that hand and Full-Body SA were highest for participants at Haulover, while the highest Adjusted-Body SA was observed for participants at Seawall. For Hand SA, all beach groups showed statistically significant difference in means, except pair 1 (Crandon) and 2 (Haulover) and pair 3 (Seawall) and 4 (Stewart) (Table S1). For Full-Body SA and Adjusted-Body SA, there were many statistically significant differences in means, except for groups 1 and 4 and 2 and 3 for Full-Body SA, and 1 and 2, 1 and 4 and 2 and 4 for Adjusted-Body SA. Many of the variances between beach groups were unequal.

### 3.5. Effect of Sunscreen on Full-Body SA and Adjusted-Body SA

**Figure 5:**
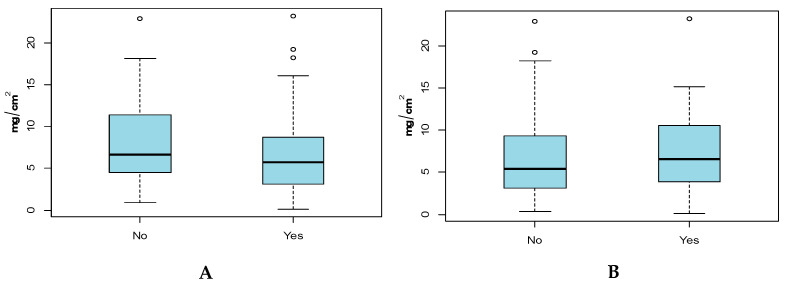
Full-body SA (**A**) sunscreen applied before play and (**B**) sunscreen reapplied during 1 h. study interval. Grey points indicate outliers.

**Figure 6:**
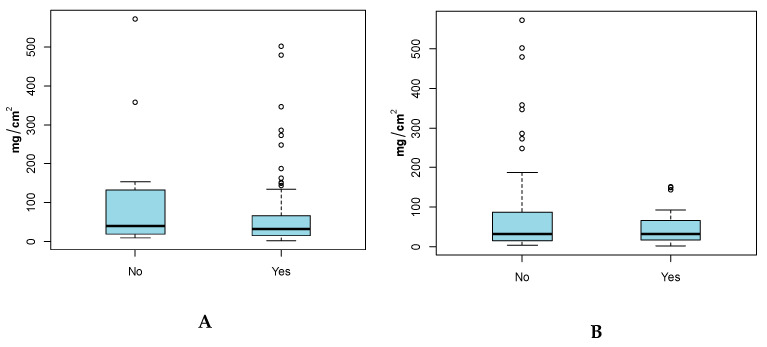
Adjusted-body SA Sunscreen (**A**) sunscreen applied before and (**B**) sunscreen reapplied during the 1 h. study interval. Grey points indicates outliers.

Results (Figure 5) show that the Full-Body SA for the group of children who applied sunscreen was less than the child group who did not apply sunscreen, although reapplying sunscreen resulted in higher Full-Body SA (although difference in means were not statistically significant) for both the first and second application of sunscreen (Table 4). Most subjects applied sunscreen before play (i.e., 99 versus 16), while only 24 reapplied sunscreen during the 1 h of beach play for Full-Body SA. Results (Figure 6) also show that the Adjusted-Body SA for the group of children who applied sunscreen was less than the child group who did not apply sunscreen, and similarly those who reapplied had lower Adjusted-Body SAs. Previously, staged hand press trials for measuring Hand SAs were performed under three conditions of testing: Dry (D), Sunscreen (S), and Wet (W), where wet hands resulted in higher adherences compared to sunscreen and dry hands, and dry hands resulted in slightly higher adherences than sunscreen hands [33]. For the D-S condition, there were no significant differences in variances and means, however for D-W and S-W conditions of testing comparisons, there were significant differences in variances as well as in mean adherences. There were some notable high numbers that can questionably be outliers, however no sound reasoning can be provided to remove them from the analysis. The four apparent outliers for Full-Body SA were three males and one female (Figure 5). For the one subject who did not wear sunscreen, his Full-Body SA was 22.89 mg/cm^2^. For the three that did wear sunscreen their Full-Body SAs were 18.20 mg/cm^2^, 19.20 mg/cm^2^, and 23.17 mg/cm^2^. Removing these outliers would drop the Full-Body Average SA from 8.83 to 8.09 mg/cm^2^ for those without initial sunscreen and from 6.36 to 5.93 mg/cm^2^ for those who used sunscreen. For Adjusted-Body SA there were a number of children that may have been apparent significant outliers in the measure of Adjusted-Body SA for wearing and especially not wearing sunscreen (Figure 6), influencing the average by having a large soil loading on the body and a small surface area over which that soil was distributed. If 5 outliers with Adjusted-Body SA’s over 300 mg/cm^2^ are removed from the overall average, average Adjusted-Body SA drops from 69.91 to 51.37 mg/cm^2^.

### 3.6. Full-Body SA Regression to Other Variables

In this section, various exploratory variables such as average adhered grain size (microns), average environmental grain size (microns), environmental humidity (%), air temperature (Celsius), water temperature (Celsius), sand temperature (Celsius) were compared in a simple linear regression against Full-Body SA and Adjusted-Body SA (Table 5). Results show that environmental grain size (microns), and environmental humidity (%) (*p* < 0.05) were correlated with Full-Body SA. Environmental grain size (microns), environmental humidity and water temperature (Celsius) were significantly correlated with Adjusted-Body SA (*p* < 0.05). R-Squared values indicated that average environmental grain size explains about 15.7% of variance for Full-Body SA. In a similar manner, average environmental humidity can explain 26.9% of variance in Full-Body SA, respectively (Table 5). It can also be seen from Table 5 that the environmental grain size can explain 18.7% of the variance in Adjusted-Body SA and humidity explains 31.9% of variance and water temperature can explain 46.9% of the variance. Adjusted R squared reduces those explanations of variance. Previous results of Hand SA also show a positive correlation with adhered grain size, average ambient grain size and water temperature (*p* < 0.05) [33].

**Figure 7:**
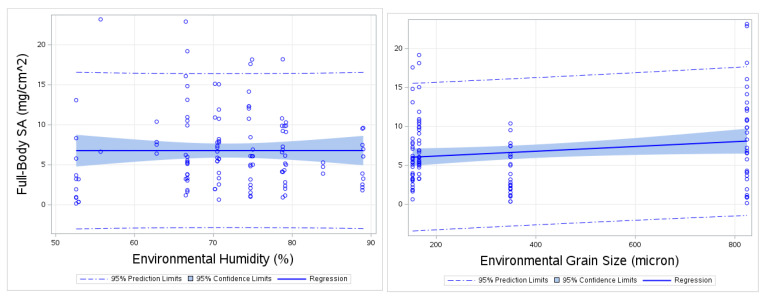
Fit plot for Full-Body SA (mg/cm^2^) compared environmental humidity (5) and environmental grain size (microns).

**Figure 8:**
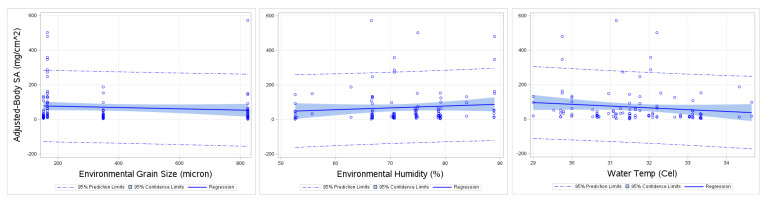
Fit Plot for Adjusted-Body SA (mg/cm^2^) compared to average environmental grain size (microns), environmental humidity (%) and water temperature (Celsius).

The plot of Full-Body SA regressed against environmental humidity and environmental grain size (Figure 7) shows slight positive slope for environmental humidity and positive slope for environmental grain size. Results showing Adjusted-Body SA regressed against environmental humidity (Figure 8), show about 6 points outside of 95% prediction limits in the fit plot. Although, this may indicate some outliers, we have no logical reason to remove these outliers. 

#### Effects of Playing with Other Children, Last Location, Last Activity, and Clothing Type on Body SA

We have also investigated the influence of other aspects of children’s play on Full- and Adjusted-Body SA, such as playing with other children, last activity and last location observed and clothing type. The majority of children were observed as documented through field notes to be playing with other children: 92 children played with other children compared to playing alone. The mean age of children who played with other children was 44.9 months, while the mean age for not playing with others was 51.3 months. Not playing with other children does not mean the children may not have played with their parents. We saw no significant difference however in Full-Body and Adjusted-Body SA for whether children played with other children or not (Table 6).

Based on the videotapes of the children, we recoded the last activity and last location of child before heading to the rinse station. One-way ANOVA tests were performed to check any significance in these activities and locations to Full-Body and Adjusted-Body SA. However, no significant difference in means for these groups was found for activity (Table 7), where the common last activity was wading (32 children). A significant difference (<0.0001) was found for last location and the Adjusted-Body Adherence where the last most common location was the dune ridge (34 children). Most children still had to walk to the rinse station however, even if last location was the seawater some sand still loads to the feet as they walk towards the rinse station with wet feet.

We also investigated the impact of clothing type on Full-Body and Adjusted-Body SA. One-way ANOVA results indicated there were no significant influence of clothing on Full-Body SA. However, some significant differences were found for Adjusted-Body SA with clothing type. Tukey’s Studentized Range (HSD) Test explored for Adjusted SA showed that clothing types of (2) bathing suit, shorts and dress or shirt, and (4) shorts, shirt and shoes where all significantly different to when the children were wearing (3) shorts (Table 8 and Table S2). Shorts had higher Full-Body and Adjusted-Body SAs (Table S3). Girls predominantly wore bathing suits and less often wore shorts only (Tables S4 and S5). The most common clothing for both males and females together corresponded to clothing type 3 (short, dress or shirt) (Tables S4 and S5). Researchers suspected that shoes worn by children into the pools for body rinses were the probable cause of higher soil loading measures. However, the Full-Body and Adjusted-Body SA were higher for children not wearing shoes (Table S6) and more influenced by higher outliers in this group.

### 3.7. Exploring the Surface Area Coverage for Various Age-Groups

There was an interest in looking at how children’s age might influence surface area loading on skin. Pictures indicated which areas of the skin had sand following one hour of play. The ratio of surface area that appeared to have sand was divided by the child’s total surface area. Higher ratios indicated more coverage of areas. T-test showed a significant difference (*p* = 0.0450) between age group 2 and 3 with age group 3 have lower ratios (Table 9). 

## 4. Discussion

In this study, we measured SA to hand and body through hand press trials and natural play. We first analyzed these SA values with respect to sex, age and beach location. Typically males had higher SAs, where this was significant for both Full-Body SA (*p* = 0.0113) and may represent their greater contact with sands through rolling and digging. The youngest age group (age 0–24 months) had higher SAs for Hand and Full-Body but the oldest age group had higher SAs. Later videotranslations over the hour of play revealed that older children spent more time wading and swimming and this may had an effect on adherence for Full-Body Adherence. Beach sites did have significant effects on Hand SA during controlled hand presses. The children that played at beaches with larger soil sizes had greater Hand SA values (i.e., Crandon and Haulover). For Full-Body and Adjusted-Body SA, this was not consistently the case. Full-body adherence did not have a consistent trend with soil sizes found at beaches, where too many factors affect play and loading in the beach setting. Adjusted-Body SA, was driven more by apparent clothing coverage on skin as documented through pictures and varied greatly.

There was an interest in seeing how other environmental conditions (temperature, humidity), along with children play, sunscreen usage and clothing might affect those adherence measures. For Full-Body and Adjusted-Body SA mean values were less for the children group who applied sunscreen before their activities on the beaches; though these differences were not statistically significant. In similar manner, for Hand SA, sunscreen use was correlated with reduced Hand SA values and this was significant in comparison to wet and dry hands. Children’s play activities were recorded through video from which the last location and activities before rinsing in the pool for Body SA measurements were extracted. However, no significant association was observed. There was significantly greater adherence observed for children wearing shorts alone, where boys predominantly wore shorts alone, most children wore bathing suits with shirts and/or dresses. This difference in clothing types may have contributed to higher adherence for boys. Later translations of videotaped data may also reveal difference in behaviors between girls and boys. Shoes worn during play or even into the pool for rinses did not have an effect on the outcomes for Body SA, where averages are influenced more by outliers (i.e., children with high loadings).

The study revealed higher adherence values for Hand SA and Adjusted-Body SA compared to many previous field studies [27,28]. This may represent the combination of large soil sizes found at these beaches, clumping of wet sand and clothing and shoes worn during pool rinses. The mean for Hand SA was 35.7 +/− 41.8 mg/cm^2^, for Full-Body SA was 6.8 +/− 4.8 mg/cm^2^, and for Adjusted-Body SA, 69.9 +/− 103.5 mg/cm^2^. The Adjusted SA shows great variability given great difference in clothing the children wore. Table 7 of the EPA Exposure Factor Handbook [23] provides a comprehensive look at geometric means for adherence by body parts by various activities across multiple studies where conditions/locations vary. The highest report adherence measure is 58 +/− 2.3 mg/cm^2^ for a child playing in mud. Previous studies have used a small number of subjects to evaluate body adherences and may not demonstrate the high variability possible in the way children play and the resulting soil loading on the body [28,30]. Soil loading may therefore then be very site-specific. Shoaf et al. [28], for example studied 9 children during 2 timed sessions (20 to 60 min) of play and at a tidal flat (i.e., similar shoreline environment) and found average geometric means for soil adherence ranging from 0.04 mg/cm^2^ to 21 mg/cm^2^ for the face, forearm, hands, lower legs, and feet. Our Full-Body SA based on natural play falls in the range. The soil in that study was described as mostly sandy, and similar to our study, however children were older at 7–12 years of age. In comparing our study with those of others, many variables differ for understanding the influence of one parameter over the other.

The greatest limitation and errors may occur with the Adjusted-Body SA measured on this project. Photos revealed obvious sand loadings, however sand may have still be present across other body parts and even on clothing areas and therefore the adjusted areas may be greatly skewing the final SA ratios. Some children (4–6 children) had high body adherences that also skewed the numbers for Full- and Adjusted-Body SA where they had either very high soil loadings or small areas that appeared to be loaded with sand. What is found on clothing and shoes from the pool rinses are not entirely a concern for dermal exposure as the skin may not be in contact with this soil loading within which contaminants are found. Suggestions for future field studies are to use the naked eye and field record areas and clothing that are soil laden. In addition, there is thought to be only a monolayer of concern for dermal exposure where contaminants in soil particles immediately above the skin are able to be absorbed during a specific time period, and dependent on the diffusion rate of the compound [40,41].

## 5. Conclusions

One third of beach visitors are under the age of 20; however, water recreation is popular within the USA population among both adults and children [42,43]. Due to their close contact to the environment and weak immunity, children of age less than 10 years are considered to be most vulnerable to adverse health effects due to biological contaminants on beaches [44,45]. Children are also more sensitive to the effects of chemical contaminants in various settings, and the same may be true of their exposure to oil spill chemicals at beaches [46,47,48]. Children express a number of unique activity patterns compared to adults that may increase their risk. In the beach environment, this may entail rolling and digging in the sand at higher rates, and practicing hand to mouth activities increasing consumption of contaminated sand [49,50]. In particular for dermal exposure, it has been shown that in neonatals and children, due to a thinner stratum corneum and greater skin hydration, there is greater absorption rates through the skin than in adults [51]. Improved estimates of sand adherence can improve the risk estimates for chemical and biological contaminants for both dermal and ingestion estimates.

In conclusion, the SA dependence on beach specific soil size, which is related to the beach location, was observed only for the controlled experiments of hand presses. There was high variability for Body SA values, representing the difference in soil condition (wet, dry) across days and beach microenvironments, and also representing the various ways in which children play. At any point during a beach visit for the child, there will be variable loading on the skin. Some sand is expected to remain on the skin until the child brushes it off or bathes in the water. Contaminated soils on the skin should be removed to reduce risk of exposure. Future work will allow us to evaluate associations in the variability (durations and frequencies of contact patterns and beach microenvironment) of play across one hour and its influence on body adherence. In addition, survey data on hygiene practices (e.g., how soon after beach play do children bathe), will offer us additional information on the residence time of sands on the skin for exposure quantification for future publications on this project.

## Figures and Tables

**Table 1:** Summary of variables.

Measures	Variable Name	*N*	Mean	Std. Dev	Median	Min	Max
Hand and Body Soil Adherence (SA)	Hand SA (mg/cm^2^)	98	35.7	41.8	19.1	0.2	234.3
	Full-body SA (mg/cm^2^)	119	6.8	4.8	5.9	0.2	23.2
	Adjusted-body SA (mg/cm^2^)	108	69.9	103.5	30.8	0.2	572.3
Sand Grain Size Measures and Pressure of Contact	Adhered Grain Size to Hands (microns)	103	464.4	348.5	314.2	147.2	1165.7
	Adhered Grain Size to Body (microns)	98	394.3	281.2	313.5	110.3	1109.6
	Pressure of Contact for Hand SA (lbs./in^2^)	97	0.8	0.3	0.8	0.2	1.7
Environmental Variables	Ambient Grain Size (microns)	122	384.0	279.8	348.9	152.2	824.2
	Ambient Humidity (%)	122	71.4	9.6	70.7	52.6	89.0
	Water Temp. (Celsius)	122	31.7	1.4	31.6	29.0	34.7
	Air Temp. (Celsius)	122	30.5	1.9	31.0	26.0	34.0
	Sand Temp. (Celsius)	118	39.4	5.5	39.0	30.5	53.5
Child Demographic	Height (cm)	122	100.8	16.4	99.1	66.0	149.9
	Weight (kg)	122	16.9	5.4	15.7	8.7	37.4
	Age (months)	122	46.5	21.2	46.0	10.0	83.0

**Table 2:** Student’s two-sample t-test for SA (mg/cm^2^) categories compared for females (F) and males (M).

SA Category (mg/cm^2^)	# of F/M	Mean	Std. Dev	Std. Err	Equality of Variances		Pooled (Equal) DF t Value *p* > |t|	Satterthwaite (Unequal) DF t Value *p* > |t|
					F Value	*p* > F		
Hand SA	F: 53	31.7	31.0	4.3	0.4	0.0005	96 −1.03 0.3040	69 −0.99 0.3237
	M: 45	40.4	51.7	7.7				
Full-body SA	F: 67	5.8	227.5	27.8	0.7	0.2478	**117** **−2.63** **0.0097**	101 −2.58 0.0113
	M: 52	8.1	5.1	0.7				
Adjusted-body SA	F: 66	56.1	86.1	10.6	0.5	0.0082	106 −1.75 0.0828	**66** **−1.62** **0.1103**
	M: 42	91.6	121.5	18.8				

#: number, bold indicates significance.

**Table 3:** Student’s two-sample t-test for SA (mg/cm^2^) categories for age.

SA Category (mg/cm^2^)	Age Groups * Compared	*N*	Mean	Std. Dev	Std. Err	Equality of Variances		Pooled (Equal) DF: t Value *p* > |t|	Satterthwaite (Unequal) DF: t Value *p* > |t|
						F	Pr > F		
Hand SA	1–2	11	77.4	81.2	24.5	5.9	<0.0001	44 2.56 0.0141	11 1.71 0.1150
		35	34.4	33.5	5.7				
	1–3	11	77.4	81.2	24.5	7.8	<0.0001	61 3.54 0.0078	11 2.00 0.0719
		52	27.8	29.0	4.0				
	2–3	35	34.4	33.5	5.5	1.3	0.3544	85 0.98 0.3283	66 0.96 0.3424
		52	27.8	29.0	4.0				
Full-body SA	1–2	27	8.3	5.6	1.1	1.55	0.2089	65 1.09 0.2790	48 1.05 0.3008
		40	6.9	4.5	0.7				
	1–3	27	8.3	5.6	1.1	1.53	0.1907	77 2.03 **0.0462**	44 1.9 0.0649
		52	5.9	4.5	0.6				
	2–3	40	6.9	4.5	0.7	0.98	0.9783	90 1.07 0.2993	84 1.07 0.2891
		52	5.9	4.5	0.6				
Adjusted-Body SA	1–2	22	69.4	124.7	26.6	1.6	0.2452	55 0.17 0.8667	37 0.16 0.8735
		35	64.3	99.5	22.3				
	1–3	22	69.4	124.7	26.6	1.6	0.1623	71 −0.17 0.8650	33 −0.15 0.8778
		51	74.0	97.8	13.7				
	2–3	35	64.3	99.5	22.3	1.0	0.8764	84 −0.447 0.6560	72 −0.45 0.6575
		51	74.0	97.8	13.7				

* Age Groups: 1 (0–24 months), 2 (25–48 months) and 3 (>48 months). Bold indicate significance.

**Table 4:** Adjusted-body SA for sunscreen applied before play (B) and reapplied after play (RA).

Measures (mg/cm^2^)	Condition	*N*	Mean	Std. Dev	Std.Err	Equality of Variances		Pooled (Equal) DF t Value *p*	Satterthwaite (Unequal) DF t Value *p*
						F Value	*p* Value		
Full-Body SA	Sunscreen (B): No	16	8.7	6.2	1.6	1.90	0.0634	113 1.82 0.0717	18 1.44 0.1665
	Sunscreen (B): Yes	99	6.4	4.5	0.5				
	Sunscreen (RA): No	92	6.5	4.8	0.5	0.87	0.6238	113 −0.80 0.4254	32 −0.77 0.4488
	Sunscreen (RA): Yes	24	7.4	5.1	1.0				
Adjusted = Body SA	Sunscreen (B): No	14	111.5	162.1	43.3	3.04	0.0019	102 1.53 **0.1292**	14 1.03 0.3190
	Sunscreen (B): Yes	90	65.6	93.0	9.8				
	Sunscreen (RA): No	83	77.2	114.6	12.6	5.52	>0.0001	102 1.05 0.2940	77 1.64 0.1097
	Sunscreen (RA): Yes	22	50.2	48.8	10.4				

Bold indicate significance.

**Table 5:** Full-Body and Adjusted-Body SA regressed against other variables.

Full-Body SA	Sum of Squares	Mean Square	Root MSE	R-Square	Adj R-Sq	F Value	*p* Value
*Average Adhered Grain Size (microns)*	2353	24	4.45	0.9368	0.1631	1.21	0.4183
*Average Environ Grain Size (microns)*	429	143	4.48	0.1567	0.1347	7.12	**0.0002**
*% Environmental Humidity*	723	38	4.51	0.2688	0.1196	1.80	**0.0306**
*Air Temp (Celsius)*	249	32	4.74	0.0947	0.0288	1.44	0.1887
*Water Temp (Celsius)*	1127	34	4.35	0.4121	0.1838	1.34	0.2510
*Sand Temp (Celsius)*	769	31	4.66	0.2844	0.0834	1.41	0.1206
**Adjusted-Body SA**	**Sum of Squares**	**Mean Square**	**Root MSE**	**R-Square**	**Adj R-Sq**	**F Value**	***p* Value**
*Adhered Grain Size–Body (microns)*	1,030,367	11,577	104.07	0.9224	0.0595	1.07	0.5084
*Avg. Environ Grain Size (microns)*	214,184	71,395	94.69	0.1868	0.1633	7.96	**<0.0001**
*% Environmental Humidity*	365,408	18,270	94.75	0.3187	0.1621	2.03	**0.0128**
*Air Temp (Celsius)*	82,971	10,371	103.65	0.0724	−0.0026	0.97	0.4675
*Water Temp (Celsius)*	537,605	16,800	90.11	0.4689	0.2422	2.07	**0.0052**
*Sand Temp (Celsius)*	355,436	14,217	100.31	0.3117	0.0911	1.41	0.1264

*p* is probability that simple linear model fits better than base line model (alpha = 0.05). R-Squared and Adj-R-Squared reflect variation in dependent variable predicted by independent variables. Bold indicates significance.

**Table 6:** Body SA compared to children playing with other children (1: Yes, 2: No).

SA Type (mg/cm^2^)		*N*	Mean	Std. Dev	Std. Err	Equality of Variances		Pooled (Equal) DF t Value *p*	Satterthwaite (Unequal) DF t Value *p*
						F Value	*p* Value		
Full-body SA	Yes	92	6.6	4.9	0.5	1.01	0.7992	116 −0.62 0.5350	42 −0.64 0.5253
	No	27	7.5	4.7	0.9				
Adjusted Body SA	Yes	83	69.9	106.0	11.6	1.16	0.7179	105 −0.05 0.9608	40 −0.05 0.9594
	No	25	70.1	96.7	19.3				

**Table 7:** Full-body SA compared to last activity observed in the videotapes (Digging, Not-In-View Running Sitting Standing Wading Walking) and last location (Back BeachTrough, Berm_Crest, Dune_Ridge, Interdial, Not-In-View and Seawater).

SA Type		DF	Mean SA (mg/cm^2^)	Sum of Square	Mean Square	Root MSE	R-Squared	Coeff Var	F Value	*p* Value
Last activity	Full-Body Adherence	6	6.71	251	42	4.70	0.0938	70.02	1.90	0.0875
	Adjusted-Body Adherence	6	70.42	119,917	19,986	101.75	0.1047	144.50	1.93	0.0832
Last Location	Full-Body Adherence	6	6.76	280	47	4.74	0.1026	70.08	2.08	0.0617
	Adjusted-Body Adherence	6	65.98	236,486	39,414	85.61	0.2477	129.75	5.38	**<0.0001**

**Table 8:** ANOVA Test for comparison of Full-Body and Adjusted-Body SA against clothing: (1: bathing suit, 2: bathing suit, short, dress or shirt 3: short, dress or shirt, 4: short, shirt and shoes 5: short, 6: diaper, 7: Full-Body clothing).

Title	DF	Mean SA (mg/cm^2^)	Sum of Square	Mean Square	Root MSE	R-Squared	Coeff Var	F Value	*p* Value
Full-Body SA	6	6.77	138	23	4.82	0.0505	71.09	0.99	0.4339
Adjusted-Body SA	6	69.91	154,463	25,744	99.11	0.1347	141.77	2.62	**0.0211**

Bold indicates significance.

**Table 9:** Ratios of Adjusted- surface areas to Full-Body area.

Title	*N*	Mean Ratios	Std. Dev	Std. Err	Equality of Variances		Pooled (Equal) DF t Value *p*	Satterthwaite (Unequal) DF t Value *p*
					F Value	*p* Value		
**1–2**	23	0.242	0.141	0.029	0.47	0.0686	57 −0.76 0.4502	57 −0.82 0.4136
	36	0.279	0.204	0.034				
**1–3**	23	0.242	0.141	0.029	0.61	0.2088	72 1.16 0.2476	54 1.27 0.2065
	51	0.193	0.180	0.073				
**2–3**	36	0.279	0.204	0.034	1.29	0.4053	85 2.08 0.0399	69 2.04 **0.0450**
	51	0.193	0.180	0.073				

## Supplementary Materials

The following are available online at https://www.mdpi.com/1660-4601/17/12/4196/s1, Table S1. Student’s Two-sample t-test for SA (mg/cm^2^) Categories for Beach Locations, Table S2. Tukey’s Studentized Range (HSD) Test for Adjusted-Body SA (mg/cm^2^) Compared to Clothing Type of 1: bathing suit, 2: bathing suit, short, dress or shirt 3: short, dress or shirt, 4: short, shirt and shoes 5: short, 6: diaper, 7: Full-Body clothing), Table S3. Clothing type vs. Full Body SA, Table S4. Clothing type vs. Adjusted Body SA, Table S5. Clothing Type Vs. Full Body SA by Sex, Table S6. Comparisons for Body-SA for the Children Wearing Shoes during Pool Rinses.
