# Correction: Ferguson et al. Soil, Hand, and Body Adherence Measures across Four Beach Areas: Potential Influence on Exposure to Oil Spill Chemicals. *Int. J. Environ. Res. Public Health* 2020, *17*, 4196

**DOI:** 10.3390/ijerph21060689

**Published:** 2024-05-28

**Authors:** Alesia Ferguson, Ashok Kumar Dwivedi, Esther Ehindero, Foluke Adelabu, Kyra Rattler, Hanna Rose Perone, Larissa Montas, Kristina Mena, Helena Solo-Gabriele

**Affiliations:** 1 Department of Built Environment, North Carolina Agricultural and Technical State University, Greensboro, NC 27411, USA; akdwivedi@ncat.edu (A.K.D.); eehindero@aggies.ncat.edu (E.E.); fradelabu@aggies.ncat.edu (F.A.); 2 School of Social Work, University of Arkansas Little Rock, Little Rock, AR 72204, USA; kyra.brown11@yahoo.com; 3 Department of Civil, Architectural and Environmental Engineering, University of Miami, Coral Gables, FL 33146, USA; hrp34@med.miami.edu (H.R.P.); l.montas@umiami.edu (L.M.); hmsolo@miami.edu (H.S.-G.); 4 School of Public Health, University of Texas-Houston, El Paso, TX 79905, USA; Kristina.D.Mena@uth.tmc.edu

## Error in Tables

In the original publication [1], there was a mistake in Tables 1–9 as published. Calculation errors were made in the first Table 1 on total body and adjusted body adherence causing a ripple effect for the other statistical calculations in other tables. Corrections were also made to all Supplemental Tables (Tables S1–S6). The corrected Tables 1–9 and S1–S6 appear in original publication.

## Error in Figures

In the original publication [1], there was a mistake in Figures 1–8 as published. Calculation errors were made in the first Table 1 on total body and adjusted body adherence causing a ripple effect for the other statistical calculations in all figures. The corrected Figures 1–8 appear in original publication.

## Citation Correction

38. Agarwal, P.; Sahu, S. Determination of hand and palm area as a ratio of body surface area in Indian population. *Indian J. Plast Surg.* **2010**, *43*, 49–53.

Should be revised to:

38. Wallace, A.B. The Exposure Treatment of Burns. *Lancet* **1951**, *257*, 501–504. https://doi.org/10.1016/S0140-6736(51)91975-7.

## Text Correction

There was an error in the original publication. This error was based on the wrong calculated values for total body adherence and adjusted body adherence.

A correction has been made to in the original publication: Abstract, Section 2.5 (Paragraph 3), Section 3.1 (Paragraph 2), Section 3.2 (Paragraph 1), Section 3.4 (Paragraph 1), Section 3.5 (Paragraph 1), Section 3.6 (Paragraphs 1, 2, 4 and 5) and Section 4 (Paragraphs 1 and 3).

With this correction, the order of some references has been adjusted accordingly. The authors state that the scientific conclusions are unaffected. This correction was approved by the Academic Editor. The original publication has also been updated.

## References

[1] Ferguson A., Kumar Dwivedi A., Ehindero E., Adelabu F., Rattler K., Perone H.R., Montas L., Mena K., Solo-Gabriele H. (2020). Soil, Hand, and Body Adherence Measures across Four Beach Areas: Potential Influence on Exposure to Oil Spill Chemicals. Int. J. Environ. Res. Public Health.

